# 2780. Factors associated with *Staphylococcus aureus* susceptibility to two lytic bacteriophages; Comparison of phage host susceptibility using a reference set of *S. aureus*

**DOI:** 10.1093/ofid/ofad500.2391

**Published:** 2023-11-27

**Authors:** Shinwon Lee, Stephanie Lynch, Nouri Ben Zakour, Geoffrey Coombs, Heejoon Myung, Jonathan Iredell

**Affiliations:** Division of Infectious Disease, Department of Internal Medicine, Pusan National University Hospital, Seo-gu, Pusan-jikhalsi, Republic of Korea; Westmead Institute for Medical Research, Sydney, New South Wales, Australia, Sydney, New South Wales, Australia; The University of Sydney, Sydney, New South Wales, Australia, Sydney, New South Wales, Australia; Murdoch University, Murdoch, Western Australia, Australia; Hankuk University of Foreign Studies, Yong-In, Gyung-Gi Do, Korea, Yong-In, Kyonggi-do, Republic of Korea; (2) Microbial Genomics Reference Laboratory, Centre for Infectious Diseases and Microbiology Laboratory Services, NSW Health Pathology, ICPMR Westmead, NSWAustralia (3) University of Sydney, Sydney, NSW Australia (4) Centre for Infectious Diseases and Microbiology, The Westmead Institute for Medical Research, Westmead Hospital, NSW Australia, Westmead, New South Wales, Australia

## Abstract

**Background:**

Timely access is a major concern when considering phage therapy. Using susceptible phage is critical for the outcomes of phage therapy. For timely access to susceptible phages, identifying factors associated with phage susceptibility and building a reference set (RS) to compare host ranges is needed.

**Methods:**

Phage K, which was a well-documented lytic *S. aureus* phage, and phage PBSA02, which was a new *S. aureus* phage from the Korean phage biobank, were used.

The efficiency of plating (EOP), the relative activity of phage against specific strain/host strain, and growth kinetics assays were used to determine phage susceptibility. The isolates with high lytic activity (HLA) were defined as susceptible (EOP > 1%).

The phage PBSA02 and phage K susceptible groups were compared to the non-susceptible groups to determine the associating factors of phage susceptibility.

**Results:**

We constructed a methicillin-resistant *S. aureus* (MRSA) RS using the Australian Group on Antimicrobial Resistance (AGAR) repository. We determined the predominant sequence types (ST) of MRSA in Australia in 2021; ST22 accounted for 90.1% of HA-MRSA and 5 STs for 67.9% of CA-MRSA (figure 1). Then, we randomly selected 39 AGAR MRSA isolates corresponding to each ST from each region in Australia (figure 1).

Phage PBSA02 exhibited HLA against 56.4% of the 39 RS isolates, while phage K exhibited HLA against 30.8%. Phage PBSA02 showed HLA against 100% of ST93 and ST30 isolates following ST1 (62.5%), ST22 (37.5%), ST5 (20%) and ST45 (16.7%); phage K showed HLA against 100% of ST30 and 62.5% of ST22 isolates but against none of the ST93 isolates.

Phage PBSA02 susceptibility correlated with the ST of MRSA isolates and ciprofloxacin susceptibility (table 1). Isolates from pneumonia/empyema tended to show phage PBSA02 susceptibility (table 1). Phage K susceptibility was also significantly associated with the ST (table 2).

**Figure:**
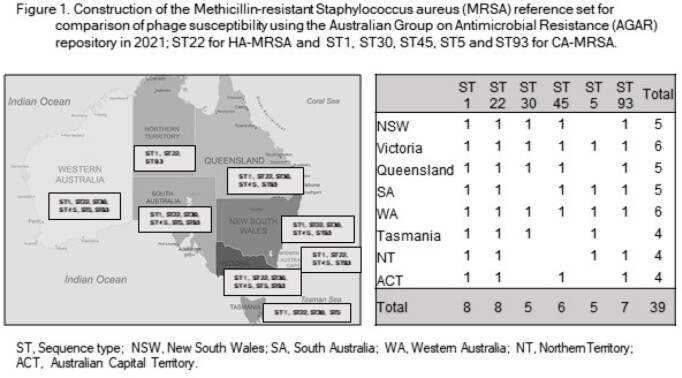

**Figure:**
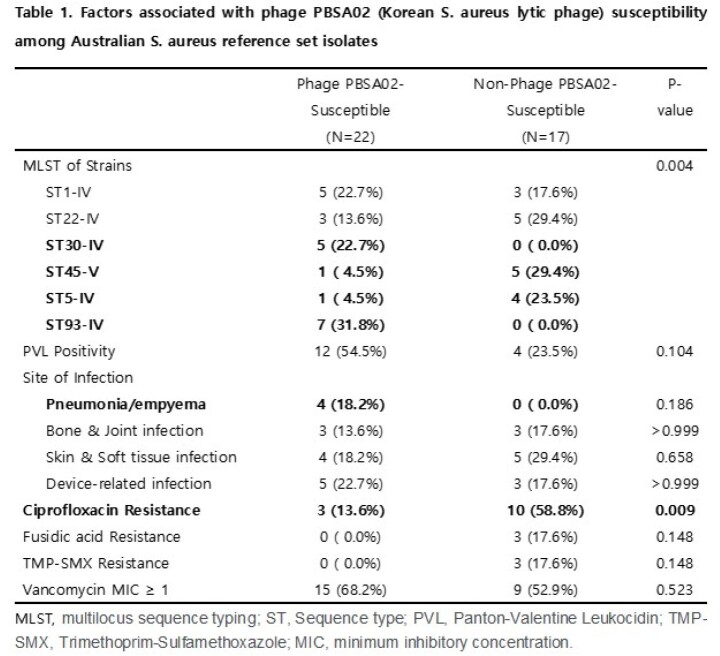

**Figure:**
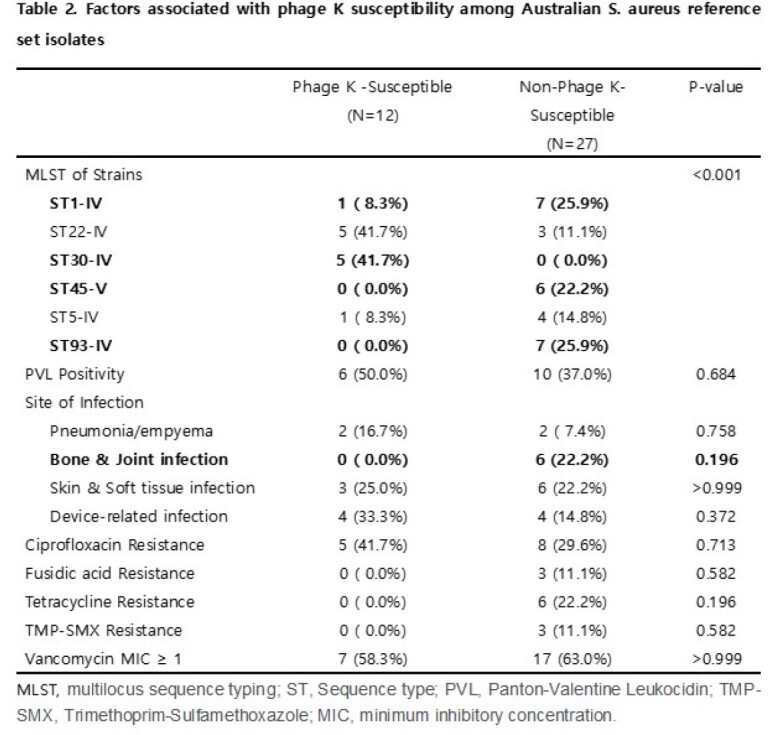

**Conclusion:**

Our study demonstrated that phage susceptibility was associated with the STs of MRSA, and the RS could be useful for a standardised comparison of the phage host range. Phage PBSA02 with broad susceptibility to the RS could be useful for phage therapy. Further work includes antibiotic synergy testing and resistance mechanism identification in these isolates.

**Disclosures:**

**All Authors**: No reported disclosures

